# Motor learning and performance in schizophrenia and aging: two different patterns of decline

**DOI:** 10.1007/s00221-024-06797-9

**Published:** 2024-03-09

**Authors:** Wouter Hulstijn, Claudia Cornelis, Anne Morsel, Maarten Timmers, Manuel Morrens, Bernard G. C. Sabbe

**Affiliations:** 1 Collaborative Antwerp Psychiatric Research Institute, University of Antwerp, Antwerp, Belgium; 2 Psychiatric Center Multiversum, Mortsel, Belgium; 3 University Psychiatric Center Duffel, Duffel, Belgium; 4 Janssen Pharmaceutica NV, Janssen Research and Development, Beerse, Belgium; 5 Donders Institute for Brain, Cognition and Behaviour, Radboud University, Nijmegen, The Netherlands

**Keywords:** Schizophrenia, Aging, Motor learning, Sensorimotor adaptation, Sequence learning, Psychomotor slowing

## Abstract

Psychomotor slowing has consistently been observed in schizophrenia, however research on motor learning in schizophrenia is limited. Additionally, motor learning in schizophrenia has never been compared with the waning of motor learning abilities in the elderly. Therefore, in an extensive study, 30 individuals with schizophrenia, 30 healthy age-matched controls and 30 elderly participants were compared on sensorimotor learning tasks including sequence learning and adaptation (both explicit and implicit), as well as tracking and aiming. This paper presents new findings on an explicit motor sequence learning task, an explicit verbal learning task and a simple aiming task and summarizes all previously published findings of this large investigation. Individuals with schizophrenia and elderly had slower Movement Time (MT)s compared with controls in all tasks, however both groups improved over time. Elderly participants learned slower on tracking and explicit sequence learning while individuals with schizophrenia adapted slower and to a lesser extent to movement perturbations in adaptation tasks and performed less well on cognitive tests including the verbal learning task. Results suggest that motor slowing is present in schizophrenia and the elderly, however both groups show significant but different motor skill learning. Cognitive deficits seem to interfere with motor learning and performance in schizophrenia while task complexity and decreased movement precision interferes with motor learning in the elderly, reflecting different underlying patterns of decline in these conditions. In addition, evidence for motor slowing together with impaired implicit adaptation supports the influence of cerebellum and the cerebello-thalamo-cortical-cerebellar (CTCC) circuits in schizophrenia, important for further understanding the pathophysiology of the disorder.

## Introduction

Schizophrenia is a disorder with a multitude of positive, negative, cognitive, mood and motor symptoms (Tandon 2022). The significance of the latter category, including motor abnormalities (e.g. neurological soft signs, catatonia and extrapyramidal signs) and psychomotor slowing, which has been defined by Osborne et al. (2020) as a measurable reduction in the initiation, amount, or speed of movement, has become apparent by an increasing number of studies conducted over the last 3 decades (Morrens et al. 2007; Walther et al. 2020; Osborne et al. 2020; Hirjak et al. 2021a, 2021b). These studies have revealed that motor signs and symptoms may serve as prodromal warning signs for a first psychotic episode (Hirjak et al. 2018) and may predict the clinical course of the illness and recovery (Cuesta et al. 2014, 2018; Osborne et al. 2020; Hirjak et al. 2021a; Nadesalingam et al. 2022). In other words, understanding motor symptoms in schizophrenia has great clinical importance.

Behavioral and biological research into these motor symptoms has been accelerated by the addition of a “sensorimotor domain” to the Research Domain Criteria (RDoC) Initiative of Mental Health (NIMH) framework in January 2019 (Hirjak et al. 2021a). This domain also includes the modulation and refinement of actions during development and learning. While motor symptoms, psychomotor slowing in particular (see Osborne et al. (2020) for review), have been demonstrated in many studies, research of motor learning in schizophrenia is still very limited. Therefore, the main purpose of the investigation presented here was to explore the abilities and limitations of motor learning in schizophrenia. Importantly, understanding motor learning in schizophrenia may have practical implications for the study of treatment effects and for improving functional outcome. As psychomotor slowing affects the proper execution of many crucial everyday motor skills, it is important to know the possibilities and limitations of training these skills. Another reason for an extensive study of motor learning in schizophrenia is the finding that learning deficits have repeatedly been demonstrated in schizophrenia in the cognitive domain. In fact, verbal learning and visual learning are included in the MATRICS (Measurement and Treatment Research to Improve Cognition in Schizophrenia)-NIHM consensus cognitive battery (Nuechterlein et al. 2004). However not much is known about possible deficits in motor learning.

In this study, patients with schizophrenia were compared with age-matched controls, and in addition with healthy elderly individuals. On a clinical level, both patients with schizophrenia and normal aging subjects show a decline in motor and cognitive functioning. For this reason, Kirkpatrick et al. (2008) and Kirkpatrick and Kennedy (2018) argued that the early stage of schizophrenia can be considered as a period of ‘compressed aging.’ Although this hypothesis is difficult to test and studies investigating cognition related to aging have had mixed results, directly comparing the patterns of decline in motor speed and motor learning in these two groups could provide additional evidence relating to this ‘accelerated aging’ hypothesis in schizophrenia. Apart from the comparison of the effects of aging with schizophrenia on motor learning, studying the possibilities and the limitations of motor learning in the elderly is important in itself. While simple motor learning seems to be intact in the elderly, the acquisition of more complex tasks and fine motor learning has been found to decline with age (see Voelcker-Rehage (2008) for review; King et al. (2013); Bootsma et al. 2021). Furthermore, explicit not implicit sensorimotor adaptation seems to occur at a slower rate in the elderly (Heuer and Hegele 2011; King et al. 2013; Lei and Wang 2018). However, explicit cognitive processes and neurophysiological motor control mechanisms behind age related changes are still far from clear and are also in need of further study (Cirillo 2021; Hooyman et al. 2021; Semmler et al. 2021).

There are many elements involved in motor learning. A skilled motor act (e.g. a tennis serve, putting on a shirt, preparing a cup of tea or writing a digit) consists of an ordered sequence of movements, each of which must be executed with improved acuity, requiring the optimization of timing, force and trajectory. Often these acts must be tailored to moving visual objects, and adapted to changes in environmental conditions. In research on motor learning, these different aspects are studied in separate paradigms. Six categories of motor learning tasks have been proposed (Ranganathan et al. 2021): sequence learning, improving acuity (Krakauer et al. 2019), tracking, adaptation, coordination and applied tasks. The present large-scale investigation of motor learning in schizophrenia and elderly individuals was conducted incorporating five of these six categories. All five task categories were executed with the same output apparatus, a pen moved by the dominant hand on a digitizing writing tablet. In addition, while each task was designed to resemble separate learning paradigms, the basic design of most of the tasks were similar, where individuals were required to make a fast execution of a pen movement to a target out of an array of possible targets. This set up facilitated the comparison of various types of motor learning. The measurement of coordination requires the recording of at least two separate movements, and was therefore not possible in the hospital setting of this investigation, which employed movement recording of a single pen. All of the tasks that were tested in this investigation are listed in Table 1.

**Table 1:** Learning tasks in the large-scale investigation

Motor learning category	Instruction	Task	
Improving acuity		Single-Aiming Task^a^	SAT
Sequence learning		Implicit Pattern Learning Task	IPLT
	Explicit	Explicit Pattern Learning Task^a^	EPLT
Adaptation		Rotation Adaptation Task	AdapR
		Gain Adaptation Task	AdapG
	Explicit	Vertical Reversal Task	VRT
Tracking		Circle Pursuit Task	PursuitC
Tracking + sequence		Figure Pursuit Task	PursuitF
Applied (writing)		Symbol Digit Substitution Task	SDST writing
*Cognitive learning*			
Symbol-digit associations		Symbol Digit Substitution Task	SDST matching
Verbal learning	Explicit	California Verbal Learning Task^a^	CVLT

^a^Results of these learning tasks have not been published previously and are presented here

A large part of the results of this extensive investigation have been published previously. However, three tasks that have been included in this test battery and not been published yet will be presented here, namely a Single-Aiming Task (SAT), an explicit motor sequence learning task and an explicit verbal learning task. The SAT tested the improvement of acuity of a simple movement. This task was modelled after the classic Fitts task (Fitts 1954). Fitts calculated an index of difficulty (ID; Log 2[2A/W]) for these movements based on their amplitude (A) and target width (W). In the present study participants made single movements in which the length of the movement (A) and the diameter of the target (W) were systematically varied. The repetition of this short task over three sessions allowed the measurement of motor learning. Previous research on single line drawing has shown that patients with schizophrenia move much slower than control participants (Jogems-Kosterman et al. 2001; Morrens et al. 2008; Docx et al. 2012, 2013; Janssens et al. 2018), however, improvement of speed and accuracy over repetition was never studied. Based on the few tracking study results, it was hypothesized that motor learning in schizophrenia would not be diminished. In the elderly, learning in fine motor tasks has been found to be reduced and therefore the hypothesis was that the elderly would show less learning in this task.

An explicit motor sequence learning task was also conducted. In the Explicit Pattern Learning Task (EPLT) clear instructions were given to participants that targets were presented in a fixed (invisible) sequence which had to be learned (see Fig. 2) and the following target had to be discovered by trial and error. Traditionally, learning a sequence of movements, sensorimotor adaptation, increasing tracking performance or motor speed and accuracy were classified as procedural or implicit learning. They were viewed as automatic and unconscious learning of information in contrast to declarative or explicit learning which involves the deliberate purpose to learn and requires conscious awareness. However, over the last decades it has become sufficiently clear that cognitive involvement appears to be important even in so-called implicit learning paradigms such as motor sequence learning and adaptation (Krakauer et al. 2019). Nevertheless, if participants are explicitly instructed that a sequence can be learned or if they are informed that they have to adapt to specific changes in movement conditions, then the rate of learning will be affected markedly. The available experimental evidence is scarce and not clear (De Picker et al. 2014; Cornelis et al. 2015, 2016, 2022). Therefore using the EPLT allows an investigation of the contrast between previously reported results of implicit sequence learning with an explicit form of motor sequence learning. It must be noted that Krakauer et al. (2019) made a clear distinction between two forms of sequence learning, i.e. learning the correct order of discrete actions (as in preparing a cup of tea or pressing a sequence of buttons) vs the activation of muscles in a particular order for executing a single movement (e.g. a reaching movement). The first, discrete, form of sequence learning has been studied more extensively. Its most frequently used laboratory implementation, the Serial Reaction Time Task (SRTT), formed the basis for the design of the Implicit Pattern Learning Task (IPLT), and this IPLT was transformed to an explicit version in the EPLT. In the IPLT the following target is always signalled by a change in colour, in the EPLT the correct target had to be discovered by trial and error. In both tasks rapid execution of the sequence profits from some form of memory of the positions of upcoming targets. Because the EPLT is a more complex motor learning task than the SAT, it was predicted that elderly would encounter greater difficulties in learning this task (Voelcker-Rehage 2008), despite preserved cognitive abilities. A second reason to expect lower learning of the EPLT in the elderly is based on reports of deficits in performing cognitive and motor tasks simultaneously (Seidler et al. 2010).

To be able to contrast the findings of explicit motor learning with a measure of cognitive learning, the California Verbal Learning Test (CVLT) was administered. The CVLT is a neuropsychological test measuring episodic verbal learning and memory. Verbal learning has been demonstrated to be reduced in schizophrenia (Nuechterlein et al. 2004) and at older age (Kramer et al. 2020), and it was therefore expected that both experimental groups in this study will have reduced verbal learning compared with controls.

In this paper results of these last three tasks will be presented. Following these results, the second part of the Results and Discussion will summarize the combined findings of the entire investigation.

## Methods

### Participants

Thirty individuals with schizophrenia, 30 healthy controls and 30 elderly volunteers participated in the study (see Table 2). At the time of testing, individuals with schizophrenia were out-patients, with a known history of schizophrenia or schizo-affective disorder (based on DSM-IV criteria), judged to be in a stable clinical condition. The evaluation was done by a trained clinician through subject interview and medical history review. All patients were treated with antipsychotic medication for at least 6 weeks, with no more than two different antipsychotic drugs used at the same time. Patients receiving treatment with benzodiazepines and anticholinergics (including tricyclic antidepressant drugs) were excluded from participating in the study because of their documented negative effects on cognition and sedative effects. Symptom severity of patients was rated by a trained psychology assistant using the scale for the assessment of negative symptoms and positive symptoms (SANS-SAPS).

**Table 2:** Group characteristics (mean and SD) for all groups and average SANS and SAPS scores in the schizophrenia group

	Control	Schizophrenia	Elderly	S-C	E-C
*N*	30	30	30		
Sex (female—male)	10–20	10–20	10–20		
Age (years)	36.8 (8.6)	36.4 (7.8)	68.7 (5.4)		
SANS score		26.2 (18.0)			
SAPS score		12.0 (18.5)			
Education years	15.1 (2.6)	12.2 (2.4)	14.5 (3.4)	*p* < .0001	ns
ART premorbid IQ	109.8 (4.9)	102.5 (8.0)	111.7 (6.4)	*p* = .0001	ns

Age- and gender-matched control participants, as well as the gender-matched elderly participants (49–79 years of age) were recruited from the local community. They met the same exclusion criteria as the patients. They were also interviewed by a clinician to verify that they had no personal history of psychiatric disorders nor first-degree relatives with psychotic disorders and that they were not using any psychotropic medication. Since the use of alcohol and drugs could potentially influence the study data, an alcohol breath test and a urine drug screen were performed before the start of each assessment day.

All candidates provided written informed consent. This study, conducted at the University Psychiatric Hospital Duffel, Belgium, was reviewed and approved by the institute’s Ethics Committee and is registered at ClinicalTrials.gov: NCT01788436.

### Task design

Three tasks are described in this paper and were part of a large test battery. All participants performed all of the learning tasks listed in Table 1. Up to 21 days prior to the first testing session, participants were tested on the *Adult Reading Test* (ART), the *Wisconsin Card Sorting Test* (WCST), and the *Letter–Number Sequencing* test (LNS; from the WAIS-IV). Following this, there were three testing sessions (each lasting about one hour) which were carried out on day 1, day 2 and day 7. The tasks listed in Table 1 were administered in the following order: DSST, SAT, IPLT, VRT, pause, CVLT, EPLT, AdapR/AdapG, Pursuit and CVLT Delayed Recall and Recognition Test. Mean group scores on these tasks are given in Table 2 and 4 (see also De Picker et al. 2014).

### New experimental tasks

The sensorimotor tasks described here (as well as the other sensory motor tasks in this test battery previously reported) employed the same digitizing writing tablet (WACOM 1218RE). Participants manipulated a non-inking pen on the tablet to control a cursor visible on a vertical computer screen at the rear of the tablet.

#### Single-Aiming Task (SAT)

**Fig. 1:**
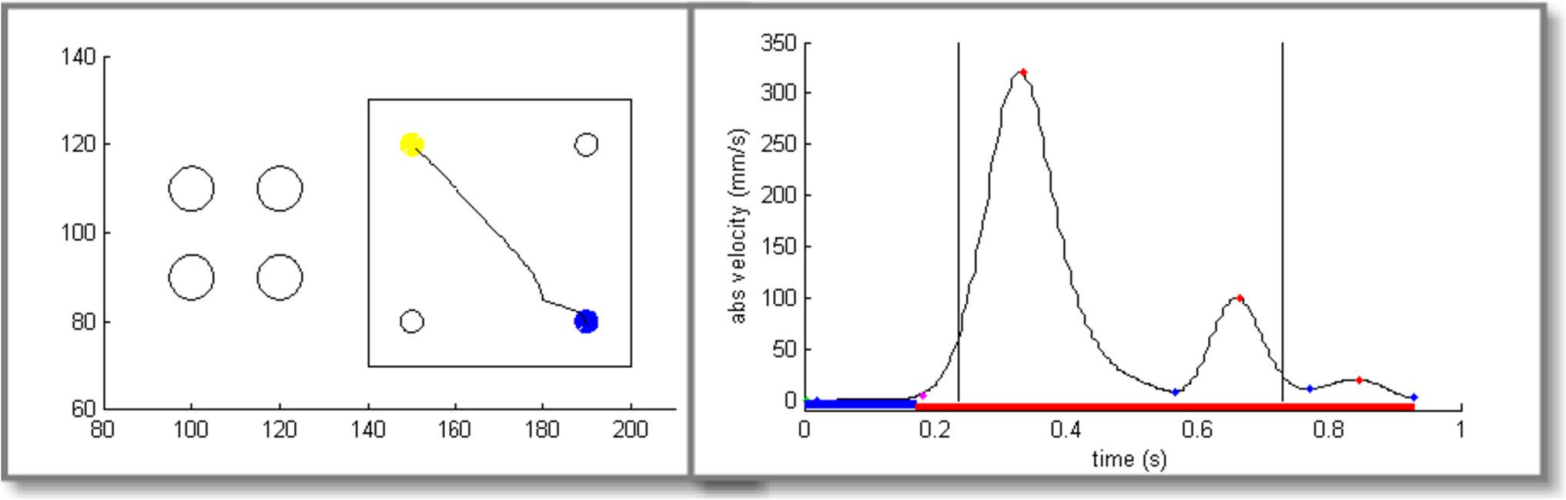
Left panel: Target display in the *Single-Aiming task* (on a 280 × 200 mm computer screen). Illustrated is an example of the cursor trajectory on a single trial of one participant. Right panel: Absolute velocity of the pen trajectory of the same participant. Peaks and valleys in velocity are marked by red and blue dots respectively, movement segments and pauses are indicated by red and blue horizontal bars. The two black vertical lines denote the crossing of the border of the yellow starting circle (defining reaction time, RT) and the crossing of the border of the blue target (defining total execution time, TT). Movement time (MT) was defined as the difference between TT and RT

This task measured improvement of speed and accuracy of a simple movement. Participants were required to move a cursor as quickly as possible to one of four possible targets displayed on a screen as open circles (see Fig. 1). The task started when the participant moved the cursor (a turquoise dot 4 mm in diameter) into the start position, which was a filled yellow circle. This immediately triggered the presentation of a target circle, a filled dark blue circle. As soon as the target was reached it changed its colour to yellow and a new blue target appeared. The trial ended when the cursor was held in the target circle for 100 ms, signalled by a short beep and a colour change of the target circle to yellow. After an intertrial interval of 100–108 ms (necessary to write data to disk) the next trial started. The order of the targets was random. A visible square border limited the possible targets to three circles (see Fig. 1, left panel). Because trials only ended when the target was reached, moving in the wrong direction resulted in longer trajectories and movement duration.

Task difficulty was manipulated by changing the distance between the circles (20 mm/28.3 diagonal or 40 mm/56.6 diagonal) and changing circle sizes (5 mm or 10 mm). Combinations of the two manipulations created four conditions increasing in difficulty (A to D). There were 2 blocks of 10 trials per condition presented in an ABCDDCBA order. With ten practice trials this 90-trial task took about 3–5 min. The very short intertrial intervals and the instruction to complete the task as fast as possible proved to be very stimulating for a rapid and accurate task execution.

The amount of motor learning in this task was assessed by measuring the improvement in MT over sessions.

#### Explicit Pattern Learning Task (EPLT)

This task measured explicit sequence learning and was similar to the SAT in that a cursor had to be moved as fast as possible from a starting position to a target. Again, as soon as that target was reached it changed colour and participants then had to move to a new target. In this task, clear instructions were given that the targets were presented in a fixed order which had to be learned. The following target was not signalled by a coloured circle as in the SAT, rather the correct target had to be discovered by trial and error. These instructions were not given in the previously reported IPLT, thus making this task a more explicit version of sequence learning.

**Fig. 2:**
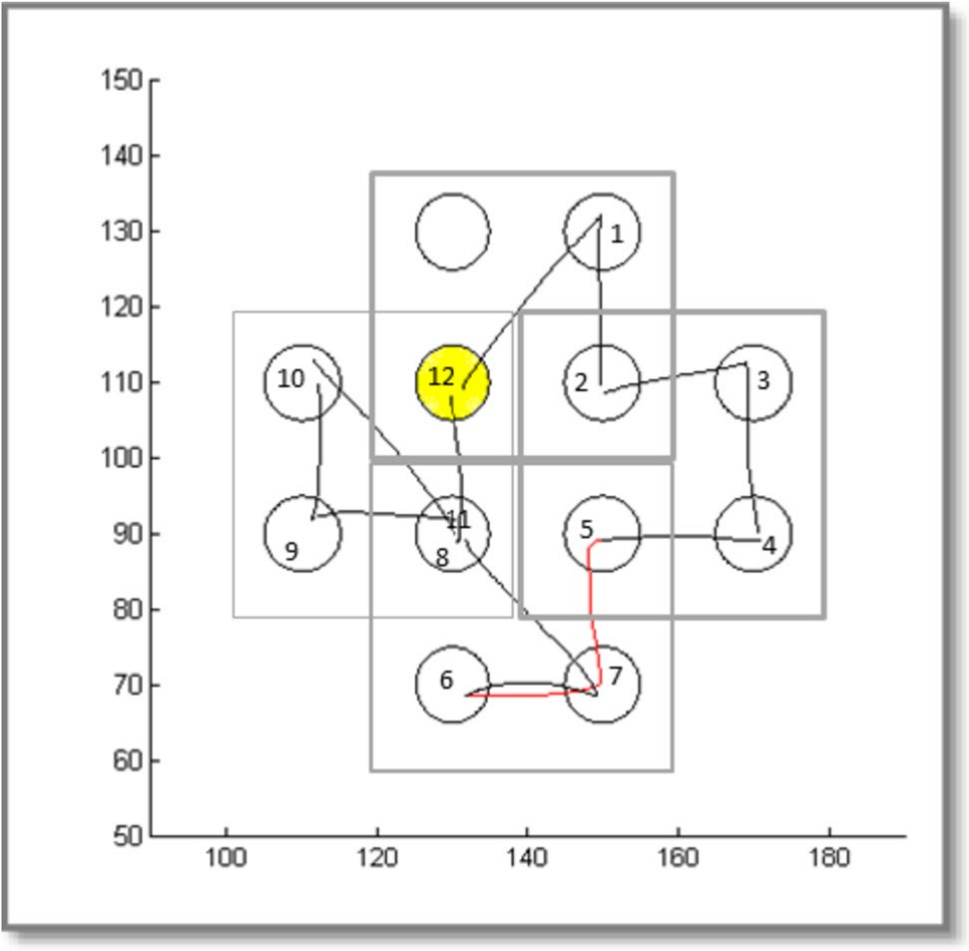
Layout of the targets display in the *Explicit Pattern Learning Task* (open circles). On the first 180 trials, a grey square was displayed indicating that the choice for the next target was limited to the other three circles within the square. The four possible grey squares shown in this figure were never presented together. The sequence that had to be learned is indicated by ascending numbers (not shown to the participants). Illustrated is the cursor trajectory made by one participant in a set of 12 trials at the end of training. This participant made one error (shown in red); on its way from target 5 to 6, target 7 was touched first

As soon as the correct target was hit, a short beep was produced, the target turned from white to turquoise, and after 100–108 ms it changed to yellow, signalling that the next target had to be discovered. Participants were told that the main goal of this task was that they should discover a fixed pattern, that it was important to make as little errors as possible and that they had to move as fast as possible from target to target. Target size (10 mm) and target distance (20 mm or 28.3 mm) was the same as in the IPLT, but to minimize transfer from the implicit version of the task, the layout of the task and the colours of the target circles were changed. On each trial a square border limited the possible target to three circles (see Fig. 2). The lines of the square border became thinner after every 60 trials and disappeared after trial number 180. As in the implicit task, the sequence that had to be learned consisted of 12 targets. Five sequences, i.e. 5 * 12 trials formed one trial block. Session 1 had five blocks of 60 trials and there were three blocks of 60 trials in sessions 2 and 3. The task was administered after the SAT and IPLT had been administered and like in those tasks the instruction and the character of the task stimulated a speedy execution, but hitting the wrong targets hindered the rapid execution of the task considerably.

#### California Verbal Learning Test (CVLT)

This task was administered as a cognitive learning task. It is a neuropsychological test measuring episodic verbal learning and memory (Kramer et al. 2020). A list of 16 words is read out to participants five times. Participants are asked immediately following each presentation to recite the words they could recollect (immediate recall; IR). Following an interval of 20–25 min (delayed recall; DR) they are again asked to reproduce as many words as possible from the list. After the DR condition, a list of 32 words is read out to them from which they are asked to identify the 16 words of the test list (word recognition; RC). In the second and third session the test list was not presented so there was no immediate recall test; only delayed recall and word recognition were tested.

### Summary of additional previously reported tasks

Implicit sequence learning was measured with the *Implicit Pattern Learning Task* (IPLT; Cornelis et al. 2016). Participants had to reach a number of targets as quickly as possible. The targets were laid out in a pattern but no learning instructions were given and participants were not informed about the repeated sequence. There were random trials (R1 and R2) and learning blocks (L1-L5).

Adaptation tasks were administered using the *Rotation Adaptation Task* (AdapR) and the *Gain Adaptation Task* (AdapG) (Cornelis et al. 2022). Participants had to make fast pen/hand upward movements towards a target as quickly as possible, but the visual movement feedback was unexpectedly altered (rotated or shortened). In the AdapR, the cursor position on the screen was rotated 30° clockwise, which forced the participants to redirect their movements counter clockwise. In the AdapG cursor movement was reduced by a factor of 0.7 which caused undershooting and required participants to make a much larger (1/0.7) movement to reach the target. Baseline trials with normal feedback were compared with adaptation trials and post-adaptation trials. The *Vertical Reversal Task* (VRT) (Cornelis et al. 2022) measured more explicit adaptation as participants were fully informed about the change in visual feedback, namely that feedback on vertical movements was reversed 180°. This required a kind of mirror drawing.

Tracking was measured using the *Circle and Figure Pursuit Tasks* (De Picker et al. 2014). In these tasks, participants had to track a continuously moving circular target with a cursor controlled by a non-inking pen on a writing tablet. The target either moved on a predictable circular path or followed an invisible trajectory (forming a complex figure).

**Fig. 3:**
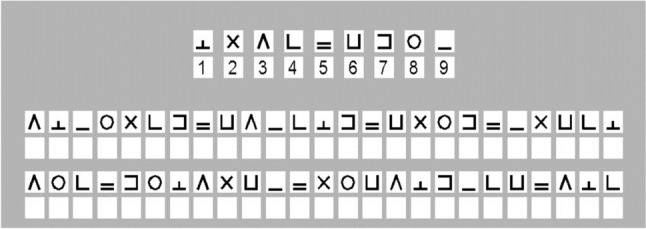
Upper part of the DSST coding sheet

An applied writing task was administered, the *Symbol-digit Substitution Task* (SDST, Cornelis et al. 2015), which is a test of psychomotor speed. Nine different symbols had to be matched to the digits 1–9, according to a key presented on top of the coding sheet (see Fig. 3). Motor speed was measured, i.e. the time taken to write each digit (writing time) as was speed of cognitive processing, i.e. the time to decode the symbols into their corresponding digits (matching time). The nine symbols were presented in random order in blocks of 9, also allowing the measurement of short-term cognitive learning by calculating the mean matching and writing time per block.

### Kinematic data

Pen movements were recorded at 200 Hz and 0.2 mm spatial accuracy. Analysis software was written in MATLAB 7.8.0. MT was the main dependent variable, defined as the time from crossing the border of the starting circle until crossing the border of the target circle (see also Fig. 1, right panel). On each trial, movements had to end in the target circle to start the next trial. Therefore, wrong or inefficient trajectories resulted in a prolonged movement duration. An error was scored when the wrong target was hit. Peaks and valleys in absolute velocity over time were used to segment the entire movement of a trial into a primary movement and any additional submovements. The first minimum in absolute pen velocity after the maximal peak velocity was used as the end of the primary movement (see Fig. 1, right panel).

### Statistical analysis

Due to technical errors, the data of a few participants (n ≤ 4) are missing in some tasks. Specifically, the number of missing participants in each task (presented in order of controls vs schizophrenia vs elderly groups) are: SAT (3 1 0), IPLT (0 1 0), EPLT (0 1 0), AdapR (1 1 0), AdapG (2 2 0), VRT (0 1 0), PursuitC (0 0 0), PursuitF (0 0 0), SDST (0 0 0), CVLT (0 1 0), WCST (1 0 2) and LNS (1 0 0). All data were analyzed with analyses of variance based on a multivariate approach (SPSS version 28, General Linear Model, Repeated Measures, of which Wilks’ Lambda test is reported). Conditions, blocks or sessions were the within-subject factors and groups the between-subject factor. Group differences of the control group with the schizophrenia group and of the control group with the elderly were tested with planned simple contrasts. Bonferroni post hoc analysis was used to compare the schizophrenia with the elderly group. Glass’ delta was chosen to characterize the effect size of group differences because the variance of data in the schizophrenia group was relatively high on some of the dependent variables. In all between-subject analyses Levene’s test for equality of error variances was administered. If Levene’s test was significant then post hoc group differences were tested with Tamhane’s T2 test and the ‘corrected’ *p* values are presented in Table 4. Alpha was set at 0.05.

## Results

### Single-Aiming Task

In this task participants made single movements towards either a wide or a narrow target (W: 5 or 10 mm) which were presented at either a short (A: 20/28.3 mm diagonal) or a long (40/56.6 mm diagonal) distance. According to the formula promoted by Fitts (1954), the ‘Index of Difficulty’ (ID) of these eight combinations ranged from 2.0 to 4.5. Equal ID (3.0 or 3.5) was obtained for two conditions: (1) a short distance with a small target (W = 5 mm) and (2) a long distance with a large target (W = 10 mm).

**Fig. 4:**
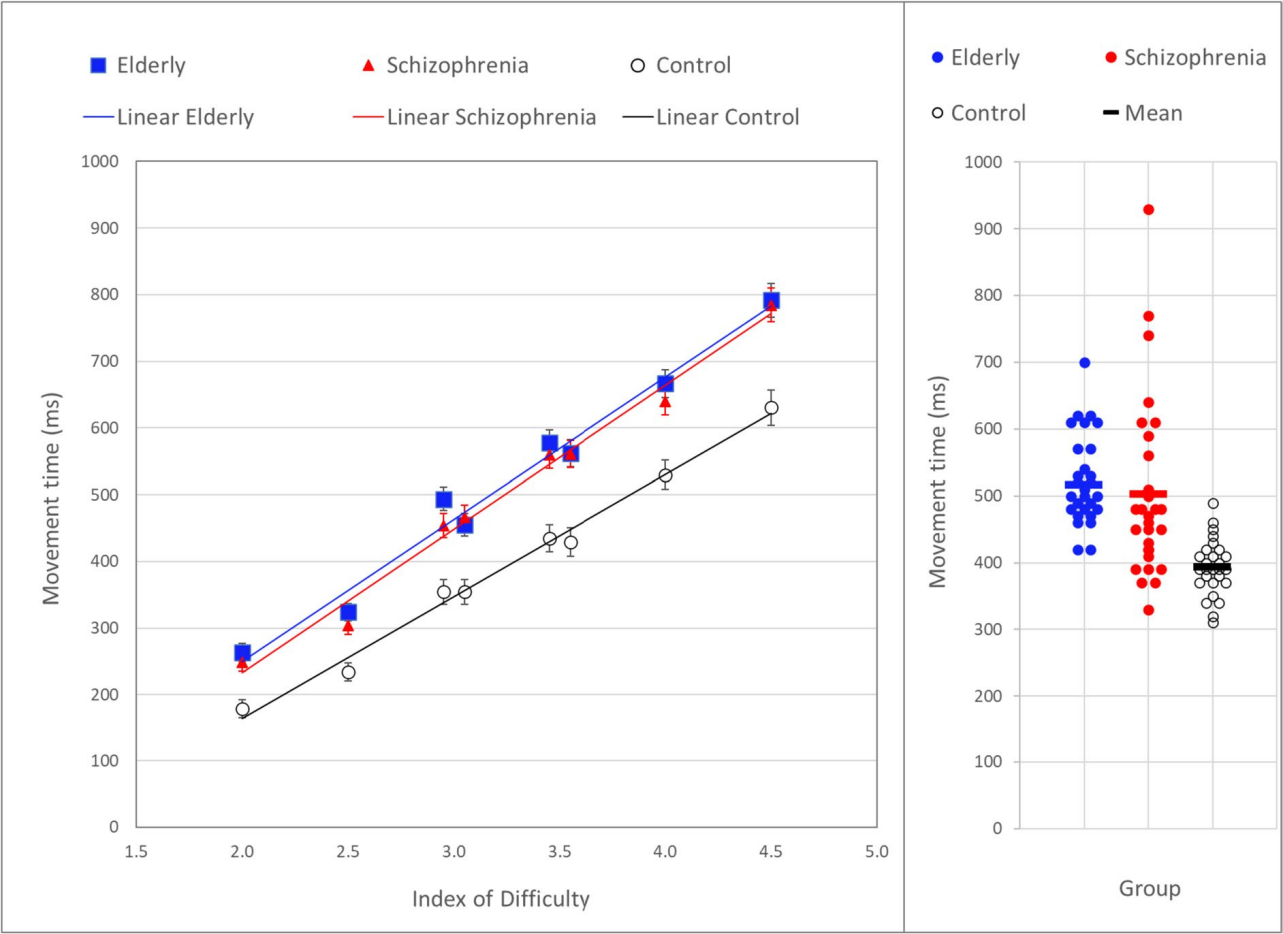
The left panel depicts means and standard error of MT per group in the *Single-Aiming Task* averaged over sessions and displayed as a function of the ‘Index of Difficulty’ (ID) of the task conditions. On the ID’s of 3.0 and 3.5, the means of the small-target conditions are presented left from the large-target conditions. The right panel presents the univariate scatterplots of the MT of each individual averaged over the eight ID conditions and averaged over the three sessions

The mean MT per group averaged over sessions for all eight possible conditions is plotted against their index of difficulty in Fig. 4 (left panel). Results of an analysis of variance on these values are presented in Table 3. Overall, controls (C) were significantly faster than both individuals with schizophrenia (S) and the elderly (E), and the schizophrenia and the elderly groups were not significantly different from each other. Figure 4 also shows that straight lines fitted these group means remarkably well (*R*^2^ ranged from 0.987 to 0.994). Slopes were also calculated for each participant and the ANOVA results on these slopes (Table 3) showed that the mean slope of the schizophrenia group (216 ms/ID) did not differ significantly from the mean slope of the elderly participants (214 ms/ID), and that both were significantly larger than the mean slope of the controls (184 ms/ID).

**Table 3:** Single-Aiming Task, ANOVA results

	Condition		Condition * Group		Group		S-C	E-C	S-E
	*F*(7,77)	*p*	*F*(14,154)	*p*	*F*(2,83)	*p*	*p*	*p*	*p*^a^
MT	366.71	<.001	2.83	<.001	14.95	<.001	<.001	<.001	1.000
Slope MT/ID					6.74	.002	.002	.002	1.000

	Session		Session * Group		Group				
	*F*(2,82)	*p*	*F*(4,164)	*p*	*F*(2,83)	*p*			
MT	126.91	<.001	4.65	.001	14.95	<.001	<.001	<.001	1.000
PM MT	6.24	.003	0.65	.630	9.70	<.001	<.001	.003	.597
SecM MT	93.71	<.001	3.05	.018	6.33	.003	.038	<.001	.461
PM in target	90.01	<.001	0.56	.694	4.75	.011	.926	.010	.032
N Sec Moves	71.17	<.001	1.73	.147	4.63	.012	.591	.006	.066
Peak Vel	45.68	<.001	0.64	.638	14.81	<.001	<.001	<.001	1.000
EM distance	100.76	<.001	0.56	.694	2.47	.091	.389	.031	.544

*MT* movement time, *ID* index of difficulty, *PM* primary movement, *SecM/Sec Moves* secondary movements, *Peak Vel* peak velocity, *EM distance* distance from end of primary movement to target

^a^Bonferroni correction

The results presented in Fig. 4 and Table 3 could give the wrong impression that the elderly and the schizophrenia groups do not differ much from each other. However, a univariate scatterplot of individual mean MT’s, i.e. averaged over the eight conditions and over the three sessions (Fig. 4 right panel) shows that this idea is incorrect. Group means of MT (E: 517, S: 502, C: 393 ms) are similar for the elderly and schizophrenia groups, but the distribution of individual mean MT’s over condition and session in the schizophrenia group is much larger than in the elderly group (SD in E: 72, S: 134 ms; Levene’s Test for Equality of variance *F* = 5.48, *p* = 0.023). In addition, also the elderly and control groups have different variances (SD in C: 42 ms, Levene’s Test *F* = 6.02, *p* = 0.017). The larger variance in the schizophrenia group is a characteristic that is also present in some other group differences presented in Table 3 and 4.

**Table 4:** Summary of test and task results on first session

	Group effect in ANOVA’s			Group means			Glass’s delta		Group contrasts	
	*F*	*df*	*p*	Control	Schizophrenia	Elderly	C S^a^	C E^b^	E S^c^	S E^d^
*Motor learning*										
Improving acuity SAT (ms)	9.83	2,83	<.001*	68	127	142	−1.28	−1.59		
Sequence learning IPLT (ms)	0.28	2,86	.754	52	51	46				
Sequence learning EPLT (Error %)	6.15	2,86	.003*	.291	.355	.570		1.36		.082
Adaptation rotation MT (ms)	5.16	2,85	.008*	185	248	193	1.08		.074	
Adaptation gain MT (ms)	7.75	2,83	.001	50	115	59	1.05		.002	
Adaptation reversal MT (ms)	11.31	2,86	<.001	544	1041	829	1.67	.96	.046	
Tracking circle (accuracy)	20.17	2,87	<.001	47	38	26	.69	1.64		<.001
Tracking figure (accuracy)	24.58	2,87	<.001*	59	50	33	1.11	2.57		<.001
*Aftereffects*										
Adaptation rotation (ms)	1.28	2,85	.283	84	40	50				
Adaptation gain (ms)	8.08	2,83	.001	78	−14	47	1.03		.010	
*Sensorimotor performance*										
SDST writing time (ms)	29.58	2,87	<.001	415	501	599	1.14	2.43		<.001
Single-Aiming Task MT (ms)	17.87	2,83	<.001*	433	576	601	2.98	3.49		
Block R1 IPLT TT (ms)	21.20	2,86	<.001*	480	610	627	2.02	2.28		
Baseline AdapR MT (ms)	4.47	2,85	.014*	320	405	402	1.23	1.18		
Baseline AdapG MT (ms)	7.01	2,83	.002*	283	383	369	1.86	1.60		
VRT baseline MT (ms)	9.92	2,86	<.001*	193	262	266	1.75	1.87		
*Cognitive learning*										
SDST matching time (slope)	0.52	2,78	.597	-.039	-.037	-.055				
CVLT IR (Nwords)	8.36	2,86	.005	12.2	9.9	11.2	1.31	.54	.019	
*Cognitive performance*										
WCST categories (N)	5.79	2,84	.004	4.0	3.00	2.6	.65	.91		
LNS (Adj score)	10.29	2,86	<.001	9.7	7.2	11.2	.70		<.001	
SDST matching time (s)	13.46	2,87	<.001	.99	1.44	1.10	1.89		<.001	

^*^Levene’s test of equality of error variances is significant

^a^Positive values denote lower learning or performance of group S compared to group C

^b^Positive values denote lower learning or performance of group E compared to group C

^c^p values (corrected) for group contrast of lower learning or performance of group S compared to group E

^d^p values (corrected) for group contrast of lower learning or performance of group E compared to group S

Mean MT of the two conditions with an ID of 3.0 and 3.5 (see Fig. 4) resulted in virtually the same values in the schizophrenia and control groups. Only the elderly showed a longer MT on the smaller target conditions. In an ANOVA on W (5–10 mm), ID (3.0–3.5) and groups, ID was significant (*F*(1,83) = 342.26, *p* < 0.001) and target width (*W*) was not significant (*p* = 0.131), but the group by target width was significant (*F*(2,83) = 4.02, *p* = 0.021).

**Fig. 5:**
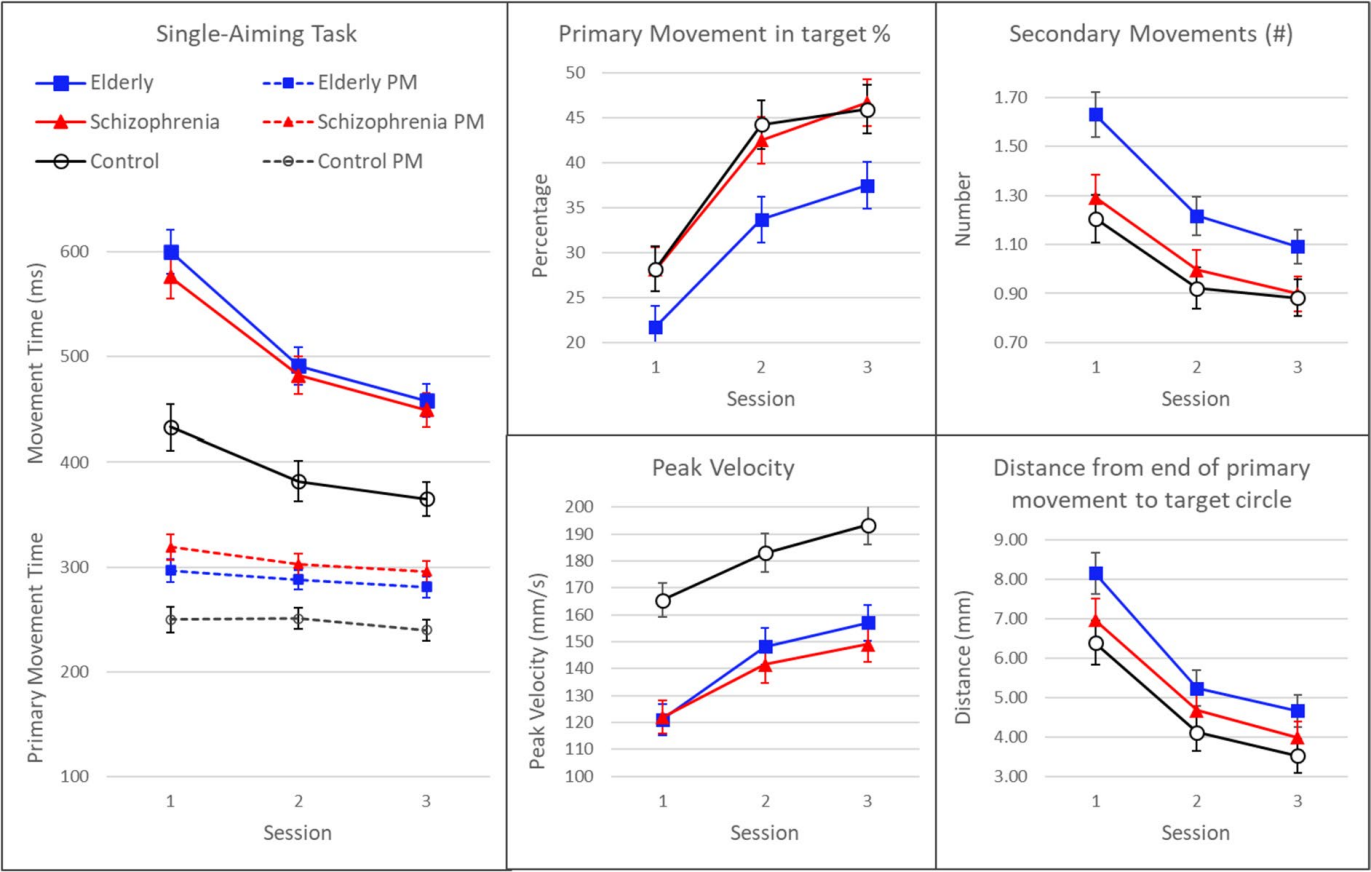
Left panel: mean and standard error of movement time (MT) and movement time of the primary movement (PM MT) per group and per session in the *Single-Aiming Task* averaged over all ‘difficulty’ conditions. Additional panels: mean and standard error of the percentage of the primary movements ending in the target (PM in target), of the number of secondary/additional submovements (N Sec Moves), of peak velocity (Peak Vel) and of distance from the end of the primary movement to the target (EM distance) of the groups per session

Although the SAT is a task that requires the execution of simple movements, all participants learned to increase the speed and accuracy of these movements. This improvement is most evident in between-session comparisons on a number of kinematic variables, which are shown in Fig. 5, averaged over the eight difficulty conditions. The largest decrease was shown by the total MT, which was about equal for the elderly and schizophrenia groups and larger than that of the control group (Table 3). This MT decrease was also calculated for each participant as the difference between sessions 1 and 3. Its group means and differences are presented in Table 4 at the label ‘improving acuity SAT’. Because this improvement (MT-decrease) was larger in the schizophrenia and elderly groups the effect sizes in Table 4 have negative values.

Total MT can be divided into the time needed to execute the main primary movement (PM MT) and the time to execute the necessary secondary movements when the primary movement did not reach the target (SecM MT). Although SecM MT is only visible in Fig. 5 as the difference between MT and PM MT, it is clear that a decrease of SecM MT made the most important contribution to the MT decrease. Although the primary MT significantly diminished over sessions (Fig. 5 left panel, Table 3), its decrease was approximately the same among the three groups, and it was much less than that of the time needed to execute the secondary movements (SecM MT). The SecM MT reduction over sessions differed between groups (the group by session interaction was significant) with schizophrenia and elderly groups showing a larger reduction than the control group.

Individuals in all of the groups were not very successful in reaching the target with their primary movement. (Fig. 5, upper middle panel). Although these percentages of successfully reaching the target increased over sessions, from 22 to 37% among the elderly and from 28 to 46% in the schizophrenia and control groups, these averages remained below 50%. This low success explains the high number of secondary movements (Fig. 5 upper right panel) with about one secondary movement per trial averaged over all trials (including those with a primary movement ending in the target). Elderly participants performed significantly worse. Their percentage of primary movements ending in targets was significantly lower and their number of secondary movements was significantly higher than the corresponding data from the schizophrenia and control groups (Table 3). On the other kinematic variables the elderly participants did not differ significantly from the schizophrenia group. This was true for the distance in mm from the end of the primary movement to the target circle (Fig. 5 lower right panel), the time needed to travel this extra distance (SecM MT averaged over sessions: E = 228, S = 196, C = 146 ms) and the peak velocity of the main primary movement (Fig. 5 lower middle panel). Together these data suggest that the accuracy of both the primary and the secondary movements was significantly lower in the elderly in comparison with the schizophrenia group.

### Explicit Pattern Learning Task (EPLT)

#### Total time to target (TT)

**Fig. 6:**
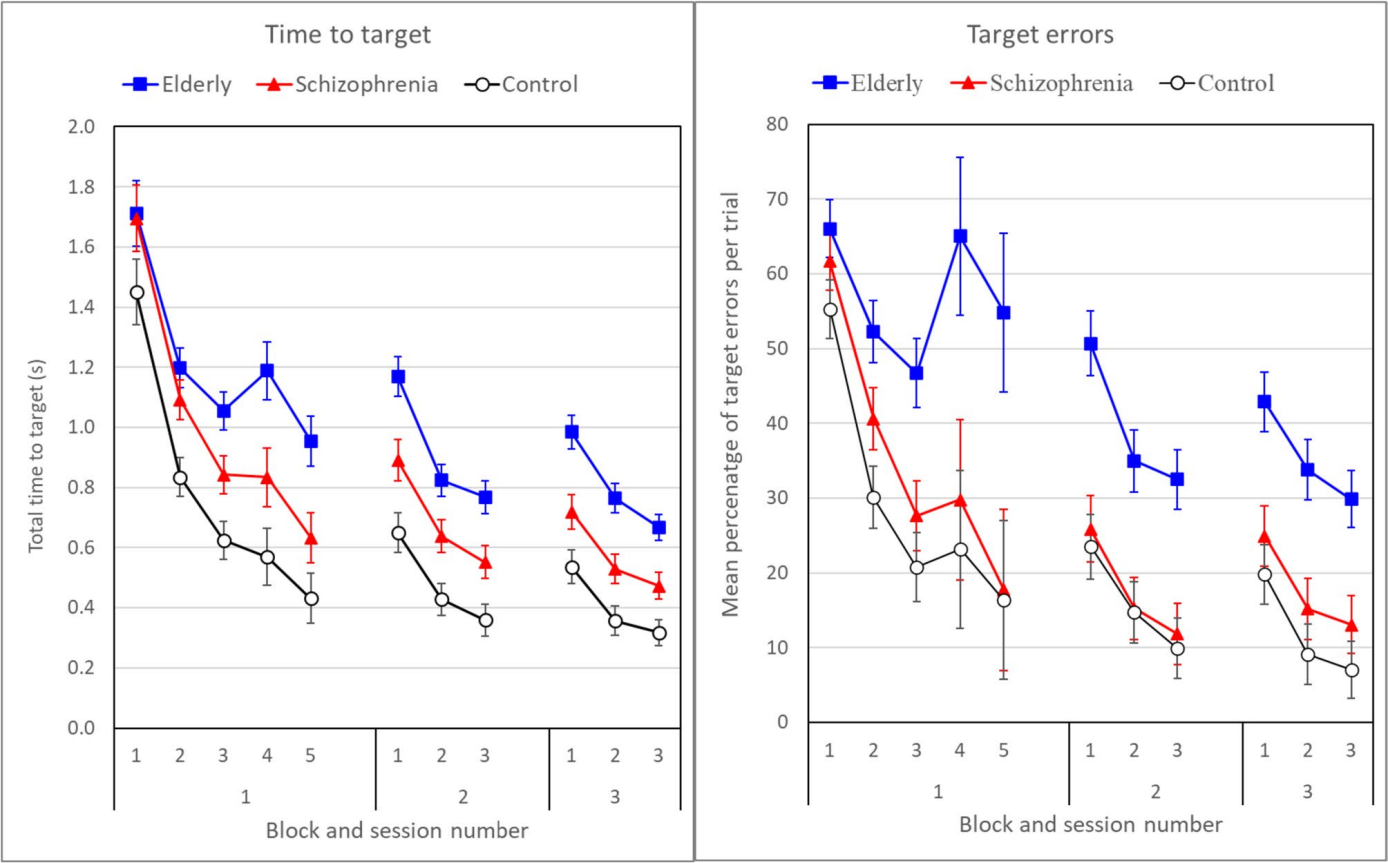
Means and standard error of total time to target (left panel) and of percentage of target errors per trial (right panel) in the Explicit Pattern Learning Task, averaged over 60-trial blocks per group

In the EPLT the target was not clearly visible but had to be discovered by trial and error. Learning in this task is most clearly expressed as the decrease of the total time needed to reach the correct target. This ‘total time’ to target (TT) is reflected by the RT, defined as the time from the start of the trial (signalled by a colour change to yellow of the starting circle) until the crossing of the border of the starting circle, added to the MT, defined as the time from RT to the time that the target circle border was crossed (as in the SAT; Fig. 1). TT was averaged over 60 trials per block and thus over 5 twelve-trial sequences. Results are displayed per group in Fig. 6 (left panel). The Figure shows a large decrease over the five blocks in session one (*F*(4,83) = 86.70, *p* < 0.001), which was similar for the three groups (block*group interaction, *F*(8,166) = 1.12, *p* = 0.355), but averaged over blocks the groups were markedly different (*F*(2,86) = 10.90, *p* < 0.001; elderly > schizophrenia: *p* = 0.037 and schizophrenia > control: *p* = 0.014). Over sessions a significant further decrease of the TT was found (*F*(2,85) = 37.97, *p* < 0.001), which was about equal for the three groups (session*group interaction, *F*(4,170) = 1.08, *p* = 0.369). In this session-effect analysis only the last three blocks of session 1 were included. Again, a significant decrease over blocks was found (*F*(2,85) = 123.07, *p* < 0.001) and no significant group by block interaction (*F*(4,170) = 1.32, *p* = 0.265). Once more the groups were significantly different on the average TT (*F*(2,86) = 17.08, *p* < 0.001; elderly > schizophrenia: *p* = 0.002 and schizophrenia > control: *p* = 0.011).

#### Target errors

Group differences in TT might be caused by less rapid learning or by lower sensorimotor speed. The percentage of target errors, i.e. actually hitting a wrong target, provides an answer (Fig. 6, right panel). On each trial a participant had to choose between three alternatives. Without learning, the percentage of errors per trial would therefore be 66.7%. This is very close to the error percentages on the first block in the first session presented in Fig. 6. From this block, the error percentages of the control and schizophrenia groups decrease sharply, while they remain high in the elderly group whereby a reduction is only seen in later sessions. In an analysis with the factors session, block and group, significant effects of session (*F*(2, 85) = 6.61, *p* = 0.002) and of block (*F*(2,85) = 22.11, *p* < 0.001) were found. In Fig. 6 error rates were averaged over the complete pattern of 12 targets. Some targets were more difficult and had higher error rates (*F*(11,76) = 13.00, *p* < 0.001), but this turned out to be equal for the three groups (group * target: *F*(22,152) = 1.20, *p* = 0.261). Most importantly, the group factor was significant (*F*(2,86) = 9.59, *p* < 0.001) but this was entirely caused by the large error rate of the elderly (elderly vs control: *p* = 0.002, elderly vs schizophrenia: *p* = 0.010) while the contrast between schizophrenia and control groups was not significantly different (*p* = 0.549). This points to slower pattern learning only in the elderly group.

#### Reaction time (RT) and summary

Additional support for this interpretation comes from splitting the TT into RT and MT. TT and MT are partly determined by the extra time needed for correcting target errors. Therefore, RT and MT were also calculated only on errorless trials. MT on errorless trials in the EPLT (E: 200, S: 211, C: 150 ms) was equal in the elderly and schizophrenia groups, but RT (E: 368, S: 288, C: 212 ms) was larger (*p* = 0.011) in the elderly group compared with the schizophrenia and control group. As a control RT was additionally calculated in the IPLT on errorless trials. In the IPLT the RT’s of the elderly and schizophrenia groups did not differ (E: 263, S: 261, C: 207 ms). Therefore the RT of the elderly stands out in the EPLT, again pointing to retarded explicit pattern learning in the elderly.

Together the results on total time to target, Target errors and RT show that explicit pattern learning was clearly retarded in the elderly group, while the rate of learning the target sequence did not differ between the schizophrenia group and the control group. Although the schizophrenia group needed a longer TT to reach the target, probably caused by their general psychomotor slowing, their decrease in TT over blocks and sessions was similar to that of the control group. In addition, their target error percentage equalled that of the control group.

### California Verbal Learning Test (CVLT)

**Fig. 7:**
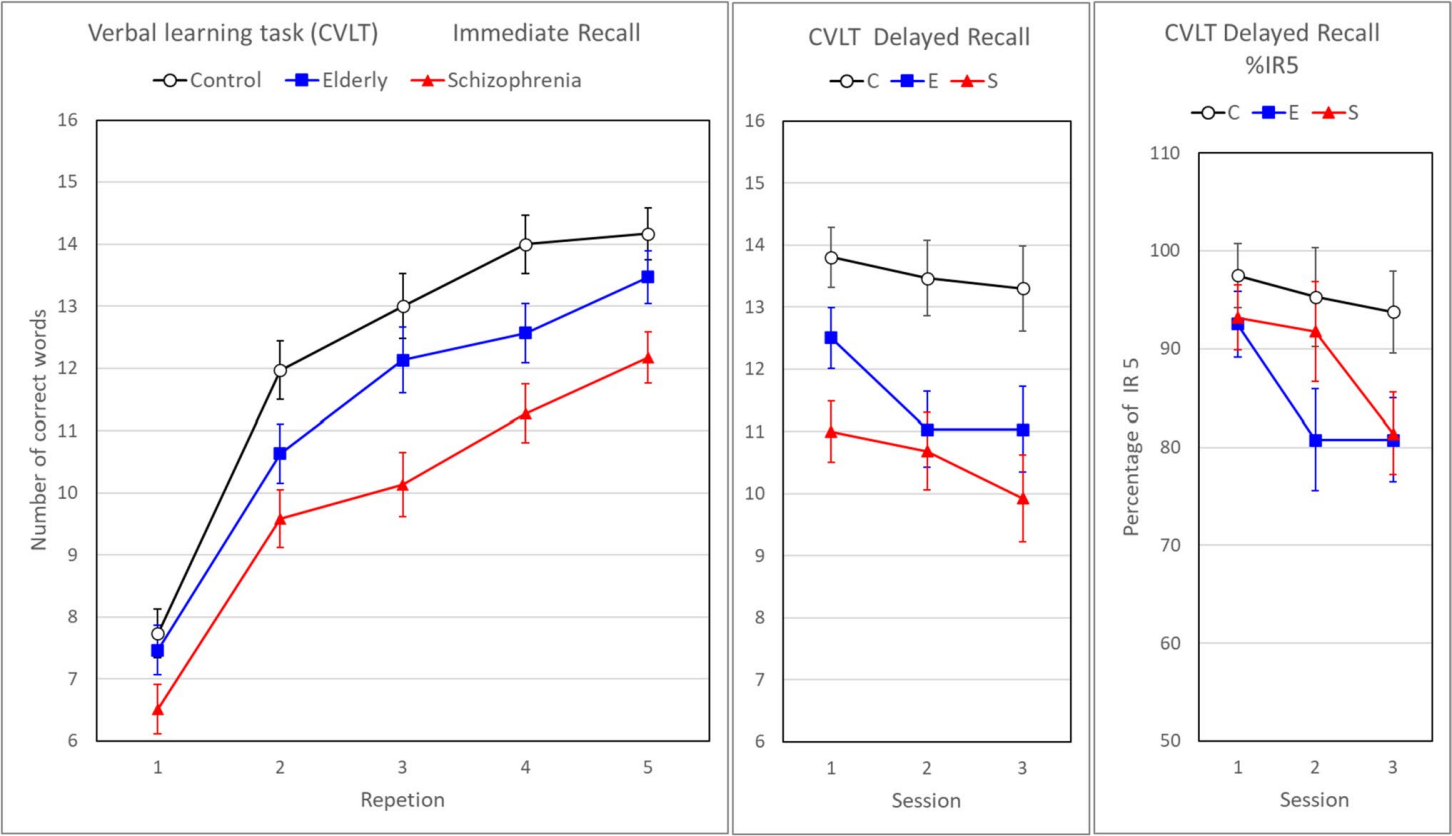
Mean and standard error of number of correct words per group over repetitions in immediate recall (left panel) and over sessions in delayed recall (middle panel). Right panel: delayed recall expressed as a percentage of the immediate recall score after the fifth repetition (%IR5)

The CVLT was administered to check for possible group differences in cognitive learning. The CVLT provides scores of the learning progress by counting the number of immediately recalled (IR) words and scores of retention in the number of words in a delayed recall (DR) test and in a word recognition test. The mean number of IR words (Fig. 7) increased over repetitions (*F*(4,83) = 161.64, *p* < 0.001) and the course of recall over repetitions was not significantly different between groups (repetition * group: *F*(8,166) = 1.73, *p* = 0.095). As expected, results from immediate recall indicated that all three groups demonstrated episodic verbal learning. However, when immediate recall was averaged over repetitions the groups differed greatly (*F*(2,86) = 8.36, *p* = 0.005). While the elderly participants were similar to the control group (*p* = 0.095), individuals with schizophrenia performed less well than elderly participants (*p* = 0.019) and controls (*p* < 0.001).

It is also quite natural that the recall of the words diminished over time and thus over sessions. Mean delayed recall (Fig. 7, middle panel) did decrease (*F*(2,85) = 11.30, *p* < 0.001) and the rate of this decrease turned out to be significantly different between the groups (session * group: *F*(4,170) = 2.28, *p* = 0.038). When delayed recall was averaged over sessions, significant group differences were also found (*F*(2,86) = 7.08, *p* = 0.001). However, groups did already differ in the number of words learned after five repetitions. Therefore, the delayed recall score was recalculated as a percentage of the immediate recall score after the fifth repetition (Fig. 7, right panel; %IR5). While this recalculation didn’t change the group by session interaction, it demonstrated that the schizophrenia group had most of the loss in delayed recall from session 2 to session 3, in contrast to the elderly group who showed only a reduction from session 1 to session 2 (Fig. 7). However, now the overall group differences were not significant anymore (*F*(2,86) = 2.13, *p* = 0.125), apart from session 3 in which the control group performed better (DR% = 94%) than the schizophrenia group (DR% = 81%, *p* = 0.04) and the elderly group (DR = 81%, *p* = 0.03). The third variable measured in this task was word recognition. It yielded high group scores, ranging from 93 to 100% of the 16 words, that were not significantly different (*F*(2,85) = 1.22, *p* = 0.301).

### Overview of previous results

**Fig. 8:**
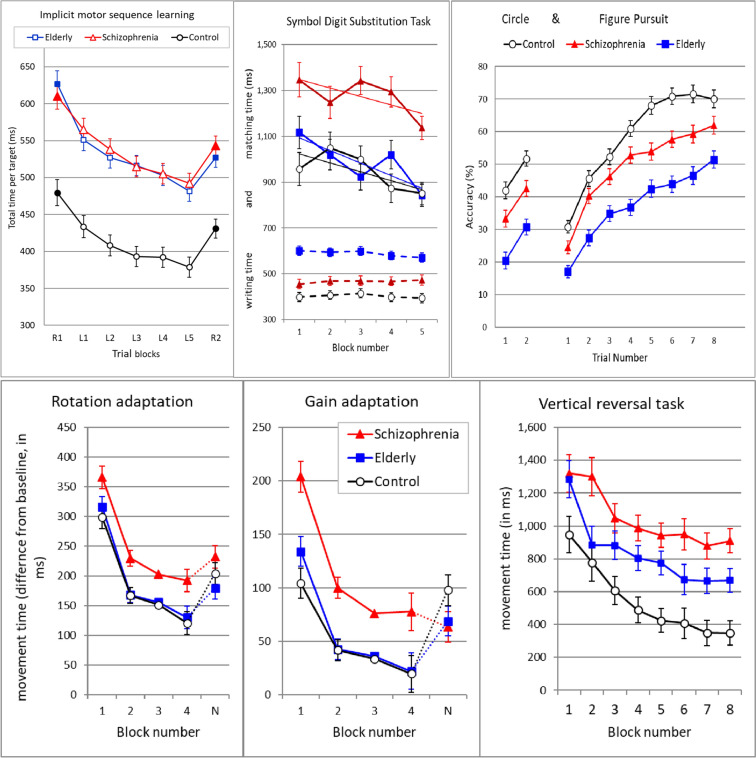
Overview of results on seven sensorimotor tasks. Illustrated are results obtained on the first session in which that task was administered

A summary of additional (previously reported) task results that were administered in this large investigation is now presented. The most essential data of these tasks are illustrated in Fig. 8. An overview of group results on motor learning and some of the aftereffects on sensorimotor performance, cognitive learning and cognitive performance is presented in Table 4. The size of the differences of the schizophrenia group and the elderly from controls is expressed as Glass’s delta scores. These are only presented when group differences were significant. The two adaptation tasks were only administered in one session, therefore the data presented in this summary are based on the first session (mostly session 1) in which these tasks were administered. The SAT learning data presented in Fig. 4 form an exception in that they were obtained by analysing the difference (decrease) in MT from session 1 to session 3. It should be noted that the data presented in Table 4 under the label ‘Motor Learning’ are not straightforward estimates of the amount of learning in these tasks. They simply show the means of MT, error percentage or accuracy over trials during learning blocks. To what extent these data validly represent the amount of learning can best estimated from inspection of the data in Fig. 8. Motor learning in EPLT sequence learning is presented in Table 4 by the mean target error percentages in session 1. Because these error percentages started at about chance level at block1, the means over all 5 blocks provide the most sensitive index of the amount of explicit learning in session 1.

### Implicit Pattern Learning Task (IPLT)

Improving acuity is visible in a decrease of total time to target (TT) from trial block R1 to R2. Sequence learning is visible from block L1 tot L5, however the most valid measure of sequence learning is provided by the difference in TT between blocks R2 and L5. Results clearly show that although the elderly and schizophrenia group were both much slower than the control group (Table 4, Block R1 IPLT), their degree of sequence learning (R2–L5, Table 4, Sequence Learning IPLT) did not differ at all.

### Rotation Adaptation Task and Gain Adaptation Task

MT and errors, i.e. initial direction error (AdapR) or the size of the undershooting (AdapG), were the main dependent variables. MTs (MT minus baseline-MT) are illustrated and ANOVA results of the mean MT over adaptation (blocks 1–4), the size of the aftereffects (MT of block N minus block 4) and the MT during baseline are presented. These analyses demonstrate inferior motor learning in rotation and gain adaptation in the schizophrenia group compared with the control group. Additionally, post-adaptation aftereffects in the AdapG provided strong evidence for impaired implicit adaptation learning in the schizophrenia group. The elderly and control groups were not statistically different. Mean baseline MT in Adap R and AdapG was rather long (around 400 ms in group S).

### Vertical Reversal Task

Figure 8 illustrates group means of MT on the eight twelve-trial blocks on which feedback of vertical movements was reversed. MT averages over these adaptation blocks (corrected for baseline MT) and over baseline trials are provided in Table 4. Data clearly show that explicit adaptation was reduced in the elderly and that this reduction was even larger in the schizophrenia group. During baseline, the schizophrenia and elderly groups were not significantly different and naturally slower than controls. An aftereffect in post-adaptation trials could was only shown in session 3 by the control group.

### Circle and Figure Pursuit Task

Accuracy with which the cursor must be held in a moving target was the main dependent variable. As expected, the control group showed the best performance, which was significantly better than the accuracy of the schizophrenia group. But unexpectedly, this latter group achieved much higher accuracy than the elderly participants.

### Symbol-Digit Substitution Task

Results show a decrease of matching time over five blocks (*F*(4,75) = 17.46, *p* < 0.001), which provides strong evidence for cognitive learning of the symbol-digit combinations. Writing time, as expected, did not change over repetitions (*F*(4,75) = 2.42, *p* = 0.056). The negative slope of matching time over blocks was calculated for each individual. Not all participants reached a score on this test of at least 5*9 matches (group S: 25, E: 28, C: 28), which explains the low degrees of freedom (*df* of 75/78) in these analyses. Results of the slope analysis (Table 4) show that the degree of cognitive learning was equal among the three groups. However, mean performance (over blocks) on the test showed large group differences. Matching time of the schizophrenia group was much larger than that of the elderly and control groups, while writing time was the highest in the elderly and the lowest in controls.

Overall, individuals with schizophrenia performed lower on nearly all items in Table 4. The exception was improving acuity in the SAT which was higher for the schizophrenia and elderly groups than for controls (expressed in negative values). This deviation can be explained from the exceptionally slow sensorimotor performance on the SAT (Glass’s delta was highest on this task). The schizophrenia and elderly groups were similarly retarded on ten tasks, the elderly performed better than individuals with schizophrenia on six tasks, while the schizophrenia group outperformed the elderly group on four other tasks.

## Discussion

A large-scale investigation of sensorimotor learning in schizophrenia and elderly participants was conducted. The current paper presents three of the learning tasks that were carried out in the two experimental groups and in healthy controls. A single aiming task (SAT) over repetitions measured improvement in motor acuity which has never been investigated in schizophrenia and elderly participants. In addition, explicit sequence learning remains unclear in both groups and was therefore investigated using the EPLT. The CVLT was administered as a measure of cognitive learning, to contrast cognitive learning and sensorimotor learning in these groups.

The large-scale investigation measured numerous sensorimotor subprocesses including explicit and implicit sequence learning and adaptation, motor acuity, tracking, applied tasks (specifically writing) and cognitive tasks. Results of the three new tasks will be discussed first. The present findings will then be discussed in light of all previous findings to map out specific deficiencies in sensorimotor learning in schizophrenia and to compare schizophrenia with aging on sensorimotor learning and performance.

### New findings

Motor learning in the *SAT* involved improving acuity, i.e. increasing speed and accuracy of a rather simple movement that varied in direction, distance and target width. Results demonstrated improvement over sessions in all three groups (MT decreased in E: 24%, S: 22% and C: 16%). This improvement was even larger for the elderly and schizophrenia group but this could be attributed to their very high initial values. While the findings that individuals with schizophrenia would not have difficulties in learning in this task were expected, the hypothesis that elderly participants would be impaired in learning in this task was not confirmed.

Interestingly, differences between the groups were found while analysing the main primary movement (PM) and secondary movements separately. In all three groups, over sessions the main PM increased in speed and accuracy and there was a decrease in the number of secondary movements. However, controls reached the targets much faster and with a higher peak velocity in the main PM than both experimental groups. In addition, accuracy of the PM was much lower in the elderly group causing them to make more secondary movements than the schizophrenia group. When comparing conditions with equal Index of difficulty (ID), a linear increase of MT with task difficulty was nearly perfect, however the elderly deviated from this straight line with a longer MT when the target was small. In other words, although the elderly showed significant learning, they were less accurate than individuals with schizophrenia.

The cause of longer MT in the elderly may be the result of slower movement speed or it may be caused by less accurate movement precision. If muscle force is more variable (less precise) in the execution of a main movement, then the target will be missed more frequently and additional movements will be needed to correct the movement, resulting in prolonged MT. This is what seems to have been the case. This finding and its interpretation is in line with earlier research suggesting that reduced movement accuracy (Voelcker-Rehage 2008) and increased movement variability (Seidler et al. 2010) requiring multiple corrective movements (Ketcham et al. 2002) is the primary cause of age-related slowing. Whether this variability originates from the planning stage of the movement or from the way these plans are transmitted by the peripheral nervous systems or executed by the muscles cannot be deducted from our data. It was observed that many elderly participants were frequently surprised and annoyed by their inaccuracy. It stimulated extra effortful attention focused on making the corrective secondary submovements as fast as possible. This attempt at compensation and the resulting cortical overactivation patterns in aging has been reviewed by Hill et al. (2020) and more recently demonstrated by Van Ruitenbeek et al. (2022).

In the EPLT participants had to learn a sequence of twelve movements. This pattern had to be discovered by trial and error. Individuals with schizophrenia were not impaired but the elderly learned this sequence slower than controls.  Unexpectedly, the rate of learning in the elderly group was much lower than that of the schizophrenia group. The high frequency of target errors made by elderly participants, even in the third session suggests that they were less successful in the storage or retrieval of the target positions.

One possible explanation for the observed difficulties in learning this task is that elderly participants might have approached this task wrongly. Despite clear instructions to put the learning of the pattern first, they may have focused too much on speed, a strategy adapted from previous tasks (such as in the SAT, among others), thus taking insufficient time between trials to consciously store the features of the positions of successive targets. This explanation would predict that the time between target movements, i.e. the RT for the next target, should be smaller in the elderly group than in the schizophrenia group. However the reverse was found. Another possible explanation emphasizes that elderly individuals require extra attention to correct their frequent movement inaccuracies. This might lower their capacity for conscious coding and storage of discovered targets while they were moving. If their goal was to find the right target quickly by trial and error and to rely on automatic storage, not spending much attention and time to store the specific features of the found target, then the high RT can be explained by trying to find in memory a location of the next target which was not properly stored earlier. In support of this explanation are many studies, reviewed by Seidler et al. (2010), that have reported deficits in older adults simultaneously performing cognitive and motor tasks.

Results of the *Verbal Learning Task* (CVLT) demonstrated that both the elderly and schizophrenia group performed less well than the control group as expected. However, in contrast to results of the EPLT, elderly participants performed significantly better on this cognitive task than those with schizophrenia. In the CVLT all attention could be focused on one type of information, while the EPLT required additional attention to movement execution. The CVLT was not administered under time pressure, and the 16 items that had to be remembered had easy (word) codes, while the twelve target positions in the EPLT required elaborate spatial coding (e.g. ‘go one step from here diagonally down to the left’). The EPLT could therefore be categorised as a complex learning task, which is in line with previous suggestions that motor learning diminishes in old age as tasks become more complex (King et al. 2013; Bootsma et al. 2021; Van Ruitenbeek et al. 2022).

Together, these new findings of two very diverse motor learning tasks and one cognitive learning task demonstrate quite different patterns of results among the groups. Specifically, elderly individuals were less accurate in the SAT and learned less in the EPLT, while individuals with schizophrenia performed worse on cognitive learning.

### Results of the current tasks in light of previous findings

Table 5 provides a comprehensive summary of all tasks conducted with their results. This summary, based on the data presented in Table 4, shows that individuals with schizophrenia and elderly individuals demonstrated significant sensorimotor learning in all categories of motor learning except in the over-learned task of writing digits. These learning results were obtained despite marked psychomotor slowing in schizophrenia and the elderly, as found in SDST writing, in the SAT and on baseline trials of the implicit sequence learning tasks and adaptation tasks.

**Table 5:** Summary of findings

	Task	Result	E vs. S
*Motor learning*			
Improving acuity	SAT	Equal learning in C S E	
	IPLT (R1-R2)	Equal learning in C S E	
Sequence learning	IPLT	Equal learning in C S E	
	EPLT	Retarded learning only in E	S better than E
Adaptation	AdapR	Retarded learning only in S	E better than S
	AdapG	Retarded learning only in S	E better than S
	VRT	Retarded learning in S and E	E better than S
Tracking	PursuitC	Retarded learning in S and E	S better than E
	PursuitF	Retarded learning in S and E	S better than E
Applied (writing)	SDST writing	No learning	
*Motor performance*			
SDST writing		Psychomotor slowing in S and E	S better than E
Single-Aiming Task		Psychomotor slowing, equal in S and E	
Baseline MT		Psychomotor slowing, equal in S and E	
*Cognitive learning*			
Symbol-digit associations	SDST matching	Equal learning in C S E	
Verbal learning	CVLT	Retarded learning in S and E	E better than S

From Table 5 it is also clear that the two experimental groups showed different patterns of results. Equal learning was found in all three groups when learning instructions were not explicit, as in improving acuity (SAT), implicit motor sequence learning and in cognitive learning of SDST symbol-digit pairs. The schizophrenia group learned better than the elderly in explicit sequence learning and in tracking, while the elderly group scored higher than the schizophrenia group on adaptation tasks and verbal learning. These patterns will be discussed below.

### Motor learning in the elderly

Elderly participants demonstrated intact motor learning on simple tasks, such as the SAT. This is supported by intact motor learning on the random blocks of the IPLT (Cornelis et al. 2016). These tasks were simple in that only one short, fast, straight movement was required towards a clearly visible target. However, on tracking tasks in which a moving target had to be closely followed, the elderly group showed significantly less learning compared to both controls and to the schizophrenia group (De Picker et al. 2014). Additionally, results from the current tasks demonstrated that explicit learning of a target sequence was more difficult for the elderly. These combined results are in line with the conclusion drawn in an often cited review by Voelcker-Rehage (2008), that in more complex tasks and with increased difficulty level, age-related learning differences become more pronounced. Furthermore, older adults rely more on visual control. This could explain why in this study elderly individuals were hardly effected by an unexpectedly altered (rotated or shortened) visual feedback of their movements on adaptation tasks (Cornelis et al. 2022). Online correction, driven by visual feedback, has become part of their normal habit.

‘Complexity’ or ‘difficulty’ of a task can be increased in different ways. Firstly, by increasing extra demands on corrective motor control, as when target size or trajectory path (Bootsma et al. 2021) become smaller or when the target is moving. Secondly, complexity increases when explicit cognitive processes needed for planning or execution of a movement sequence ask for more elaborate processing, as when sequences are longer or when the spatial coding of the targets is more complex. An account of why ‘complexity’ results in less motor learning is given in Seidler’s “Supply and demand” framework (Seidler et al. 2010; Seidler and Carson 2017). In this framework, deficits in motor performance in old age such as increased variability of movement and slowing of movement, are caused by a dysfunction of the central and peripheral nervous systems as well as the neuromuscular system. This motor deficit, meaning less supply of motor control, then requires higher demands on cognitive brain processes needed for motor control, and this in turn reduces the capacity for cognitive learning of target sequences.

### Motor learning in schizophrenia

Individuals with schizophrenia also demonstrated intact motor learning on simple tasks, such as the SAT as well as in the random blocks of the IPLT (Cornelis et al. 2022). In addition, on three implicit learning tasks (SAT, IPLT and SDST matching) individuals with schizophrenia learned as well as controls. When explicit, conscious-cognitive processing was called for, as in the EPLT, the rate of learning in the schizophrenia group was not significantly reduced compared with controls. This is in contrast with the reduced performance in the CVLT, a cognitive learning task, where individuals with schizophrenia performed worse than both controls and the elderly, highlighting their cognitive difficulties. Manifestations of a cognitive deficit interfering with motor learning tasks were observed in the implicit sequence learning task, in which subjective sequence awareness arose significantly less (Cornelis et al. 2016). Therefore, it can be suggested that individuals with schizophrenia are impaired on sensorimotor learning paradigms in which explicit cognitive processes play a role.

However, in addition to the sometimes minor effects of deficient explicit cognitive processing on learning in schizophrenia, their slower adaptation in the three adaptation tasks (see Fig. 8, Table 4) is more remarkable. They might have detected the perturbation of the movement feedback later (as argued in Cornelis et al. 2022), but on later adaptation trials they still lagged far behind the elderly and the controls in adjusting their movements to the altered sensory feedback. Even more revealing was their behaviour on post-adaptation trials, specifically in the gain adaptation and the VRT, which showed that they had not rebuild or changed an automatised forward model for movements in the altered situation, a model which needed to be corrected when normal feedback was again restored. Visuomotor adaptation is now generally viewed as the combined action of explicit learning driven by the detection of a performance error and implicit learning of a forward model driven by prediction error (Heuer and Hegele 2011). The significant different behaviour on post-adaptation trials of the schizophrenia group compared with controls and the elderly, suggests that this implicit sensorimotor adaptation in schizophrenia is also impaired. The implications of difficulties in motor adaptation in schizophrenia may suggest a general disability to adapt to changes in any situation. A suggestion of further research is evident.

### Cognitive and motor influences on sensorimotor slowing in schizophrenia

It is important to understand the nature of slow motor performance demonstrated in schizophrenia (see Table 5) and highlight that a diminished speed of cognitive processes related to actions in schizophrenia must play an important role. At a low level of cognitive processing, sensory processing (both auditory and visual) has been demonstrated to be dysfunctional in schizophrenia and found to contribute to higher-order cognitive dysfunction (Dong et al. 2023). Sensory discrimination has also been found to be significantly lower in individuals with schizophrenia (Koshiyama et al. 2021). In addition, higher order perceptual processes have been demonstrated to be deficient in schizophrenia using various drawing (copying) tasks (Jogems-Kosterman et al. 2001; Morrens et al. 2008; Grootens et al. 2009; Bervoets et al. 2014; Janssens et al. 2018). Copying rests on cognitive processes such as recognition, coding, storage in working memory and subsequent retrieval of the figure that has to be drawn. It also requires the use of executive processes to plan the optimal movement sequence. In addition, slowing in schizophrenia may arise from difficulties in monitoring the movement. It is therefore quite plausible that individuals with schizophrenia were less accurate or later to detect deviations from their planned movement. In addition, they might have been slower in making necessary movement adjustments. Monitoring and quick correction require intensive focused attention and sufficient arousal, which also might have been suboptimal in the schizophrenia group.

In a recent review on psychomotor slowing in schizophrenia, Osborne et al. (2020) made a distinction between cognitive (prefix “psycho”) and motor execution (root word “motor”) aspects of psychomotor slowing. Motor aspects were defined as processes implicated in the initiation, coordination, and execution of movements. Many studies have demonstrated that individuals with schizophrenia have impaired cognitive processes involved in response selection and motor preparation, however findings of impaired motor execution are less consistent (Osborne et al. (2020, *p* 6)). Following this, the SAT and the baseline stages of IPLT, EPLT and VRT are a step towards investigating ‘pure’ motor execution aspects of sensorimotor slowing as these tasks require minimal cognitive processes. The present study therefore provides strong evidence for ‘motor’ slowing in schizophrenia (evident with very large effect sizes, see Table 4). This evidence is consistent with previously reviewed slow movements in the line-copying task (Jogems-Kosterman et al. 2001; Morrens et al. 2008; Docx et al. 2012, 2013; Janssens et al. 2018).

Although the SAT has the least cognitive components compared with other tasks, this task still required some implicit planning involving the choice for the optimal posture of arm, hand and fingers. Similarly, drawing a single line follows several implicit planning rules or so-called graphic production rules about the best way to start and to connect lines (Thomassen et al. 1991). When drawing a series of lines that gradually tilt from vertical to horizontal, somewhere half way in that series most people change their movement direction from top-down to left–right. Individuals with recent-onset schizophrenia made this shift much less frequently or much later than healthy controls (Grootens et al. 2009). In addition, when individuals with schizophrenia were instructed to begin drawing at a point that conflicted with the preference predicted by graphic production rules, more time was needed to initiate the drawing (Jogems-Kosterman et al. 2006; Grootens et al. 2009). Together these results show that implicit planning of very simple movements is also affected in schizophrenia.

Implicit planning of a movement, such as selection and positioning of our limbs is done without awareness of the choices or the forces that are involved. Yet it is based on ‘knowledge’, and the fact that a strong learning effect was demonstrated over sessions in these tasks suggests that this ‘knowledge’ can be increased. Therefore, it is hard to draw a line between ‘psycho’ and ‘motor’ in action research, on a scale between pure motor execution and higher order cognitive processes (Rosenbaum 2017; Rosenbaum and Feghhi 2019).

### Implications of findings in schizophrenia

As argued before, a large array of different cognitive processes are closely related to sensorimotor slowing and diminished motor learning in schizophrenia. It has been proposed that the neural underpinnings of these processes comprise of parieto-frontal networks, the supplementary motor area (SMA) and pre-supplementary motor area (pre-SMA), important for planning movement sequences (Osborne et al. 2020). This view has been broadened to include effects of biochemical modulation, specifically taking into account affective changes interacting with psychomotor mechanisms leading to abnormalities (Northoff et al. 2021). In this view, the interaction between ‘psycho’ and ‘motor’ is highlighted, and a strict division of motor function from affective and cognitive function is rejected.

Difficulties in sensorimotor adaptation in schizophrenia provide evidence for the connection between these different functions on a neurobiological level. Sensorimotor adaptation relies heavily on cerebellar activity (Seidler et al. 2010; Izawa et al. 2012; Krakauer et al. 2019). An influential integrative theory of schizophrenia, already proposed by Andreasen et al. (1998), posits a cognitive dysmetria model in which a disruption to the cortico-cerebellar-thalamic-cortical circuit underlies a broad set of sensorimotor and cognitive dysfunction. In this circuit, the cerebellum plays a primary coordinative role and one way to test this theory is to examine if adaptation in schizophrenia is diminished (Cornelis et al. 2022). More recently also Mittal et al. (2021) stressed the importance of the role of the cerebellum and the CTCC circuits in psychomotor activity, which is in line with neuroimaging studies demonstrating CTCC dysfunction relating to sensorimotor abnormalities in schizophrenia (Hirjak et al 2021a). Importantly, evidence for ‘motor’ slowing found in the present study (see Table 4, Table 5 and the paragraph below), together with evidence of impaired implicit sensorimotor adaptation, strongly support CTCC dysfunction in schizophrenia.

In addition, psychomotor slowing is not an unitary phenomenon, but consists of a wide range of distinct sub-processes of specific cognitive and motor deficiencies with possible different patterns across individual patients. This has important implications for future research. Clearly making a distinction between ‘psycho’ and ‘motor’ components is not only difficult but is also simplifying and masking the variety of possible delays in sensorimotor learning and performance. On the other hand, while it is valuable to stress the interconnectedness of cognitive and motor processes (Northoff et al. 2021), treating psychomotor slowing in schizophrenia, depression and Parkinson’s disease as a uniform dimension could detract from the ultimate goal to find the underlying causes of the motor abnormalities in these illnesses, which are probably highly different. Therefore, as a supplement to the extensive research on cognitive impairments in schizophrenia, which has led to the identification of separable cognitive factors in schizophrenia (Nuechterlein et al. 2004), future research should be conducted in the motor domain (in a RDoC perspective) focusing on distinct sub-processes contributing to psychomotor slowing in schizophrenia.

In the present study it was demonstrated that rates of learning in various motor learning categories differed highly between schizophrenia and elderly. One of the aims of this investigation was to compare supposed declines in categories of motor learning in schizophrenia with expected decreases in the elderly. This was motivated by recent research of Kirkpatrick et al. (2008) and Kirkpatrick and Kennedy (2018), supporting the theory that schizophrenia might be a neurodegenerative disorder with genetic, functional-organic and neuroanatomical features of accelerated aging sharing similarities with elderly individuals. However, results of the present study demonstrating different patterns of decline in motor speed and motor learning in schizophrenia patients and the elderly do not support this hypothesis.

Findings of this study have clinical implications as well. Daily functioning relies heavily on the quality of a range of cognitive abilities and motor skills. Both individuals with schizophrenia and their therapists must realise that not only do cognitive deficits have negative influences on functional outcome but also declining psychomotor skills play a role as well, specifically on motor skills. While psychomotor slowing and learning of sensorimotor tasks in schizophrenia is more pronounced as cognitive demands increase, their difficulties are not restricted to complex, cognitively charged motor skills. Difficulties also manifest in very simple motor tasks, which may provide valuable information for daily functioning in work and home situations. In addition, variability found amongst patients suggests that psychomotor slowing may not be an obstacle for all patients and suggests the use of testing motor skills to understand their limitations and to advise on employment opportunities. The finding that significant motor learning is possible might be of value for therapeutic programs in which motor skills are trained (i.e. sport, music or other leisure activities). It is also important in training to take into account their difficulties with adaptation to changing sensory conditions.

### Limitations

A few strengths and limitations of this large scale investigation should be mentioned. Its strength lies in the design of the investigation in which multiple motor learning tasks were studied on the same set of participants and over repeated sessions. This might have created a limitation in that only individuals who were able to complete the tasks in all three one hour sessions were included in the study. As such, the motor learning and performance capabilities demonstrated in this study might be higher than what would be expected in schizophrenia and at old age respectively. However, the mean and range of scores on the negative symptoms scale of the patients in the present study were comparable to a large heterogenous sample of patients with schizophrenia (Van Erp et al. 2014), suggesting that results of the current study may be reflective of general schizophrenia. A second limitation concerns the fact that all patients with schizophrenia in the current study were taking (more than) one antipsychotic at the time of testing. The effect of antipsychotics on motor learning has still to be investigated.

It is possible that movement slowing in schizophrenia could be the result of sedentary lifestyles as opposed to neurological factors. Studies using actigraphy on patients with schizophrenia (Pieters et al. 2021), showed that low physical activity and sedentary behaviour of many of these patients is associated with movement disorders, in particular slowing evident in parkinsonism. However, the patients in the current study were out-patients and the elderly made a rather active impression on the evaluation clinician. More research is needed to determine whether psychomotor slowing leads to sedentary behaviour or whether an inactive life style results in observed psychomotor slowing.

## Conclusion

Individuals with schizophrenia and the elderly both demonstrated motor slowing but nevertheless showed significant but different motor learning abilities. The differences were apparent by impaired adaptation in schizophrenia and reduced explicit motor sequence learning in the elderly. While motor slowing in schizophrenia appears to be caused by implicit planning deficits, slowing in the elderly may be caused by less accurate movement precision. Importantly, cognitive deficits seem to interfere with motor learning in schizophrenia and task complexity interferes with motor learning in the elderly. In other words, a different pattern of decline is demonstrated.

The current study investigating sensorimotor learning in schizophrenia extends current knowledge of psychomotor slowing in schizophrenia, highlighting that psychomotor slowing is not a uniform phenomenon, rather it consists of a range of distinct sub-processes of specific cognitive and motor deficiencies with different patterns across individuals. Understanding mechanisms underlying psychomotor slowing in schizophrenia is essential to target proper training, which can improve everyday motor skills. In addition, evidence for motor slowing together with impaired implicit adaptation supports the influence of cerebellum and the CTCC circuits in schizophrenia, important for further understanding the pathophysiology of the disorder.

## Data Availability

All data generated or analyzed during this study are included in this article and/or its online supplementary material. Further inquiries can be directed to the corresponding author.
